# Survey on synergism effect of ketotifen in combination with pyrimethamine in treatment of acute murine toxoplasmosis

**DOI:** 10.1186/s41182-017-0079-0

**Published:** 2017-11-21

**Authors:** Mahbobeh Montazeri, Kian Rezaei, Mohammad Ali Ebrahimzadeh, Mehdi Sharif, Shahabeddin Sarvi, Ehsan Ahmadpour, Mohammad Taghi Rahimi, Abdol Satar Pagheh, Saeed Mehrzadi, Ahmad Daryani

**Affiliations:** 1 Toxoplasmosis Research Center, Mazandaran University of Medical Sciences, Sari, Iran; 2 Student Research Committee, Mazandaran University of Medical Sciences, Sari, Iran; 3 Pharmaceutical Sciences Research Center, School of Pharmacy, Mazandaran University of Medical Sciences, Sari, Iran; 4 Department of Parasitology and Mycology, Sari Medical School, Mazandaran University of Medical Sciences, Sari, Iran; 5 Infectious and Tropical Diseases Research Center, Tabriz University of Medical Sciences, Tabriz, Iran; 6 School of Medicine, Shahroud University of Medical Sciences, Shahroud, Iran; 7 Razi Drug Research Center, Iran University of Medical Sciences, Tehran, Iran

**Keywords:** *Toxoplasma gondii*, Ketotifen, Cell membrane stabilizer, Quantitative PCR

## Abstract

**Background:**

Standard treatment of toxoplasmosis is accompanied by severe side effects and low tolerability; accordingly, alternative medicines are critically needed. Ketotifen (KET) as a cell membrane stabilizer could be an appropriate inhibitor of *Toxoplasma gondii* (*T. gondii*) parasite entrance into the host cells. Therefore, the focus of current study is characterization of the anti-*Toxoplasma* activity of KET in the acute phase of toxoplasmosis in murine model as pre-treatment and post-treatment (before and after infection with RH strain). KET was used intraperitoneally both individually (2 and 3 mg/kg/day) and in combination with pyrimethamine (PYR) (50 mg/kg/day). One week after the post infection, DNA was extracted from brain biopsies samples. Parasite load was calculated using Quantitative-PCR (Q-PCR) in a triplicate reaction for each DNA with the target for at RE (a 529 bp repeat element) gene.

**Results:**

A significant difference between KET and control groups was observed (*P* < 0.001) in the pre-treatment and post-treatment groups. Both KET and the combination of KET and PYR showed a reduction in the parasite load in brain through the acute phase of the infection. 2 mg/kg/day dose of KET resulted in higher anti-*Toxoplasma* activity (15,698 parasites/ml) compared to 3 mg/kg/day dose of KET (72,898 parasites/ml) in brain in the pre-treatment group. In addition, KET combined with PYR significantly decreased the parasite load in the post-treatment group.

**Conclusions:**

Our results indicated that KET has both prophylactic and therapeutic effects on acute phases of the disease.

## Background

*Toxoplasma gondii* (*T. gondii*), a ubiquitous intracellular parasite, is the etiologic agent of toxoplasmosis [1]. Approximately, over 1 billion of the world population is chronically infected by *T. gondii.* This infection can be transmitted to humans by various transmission pathways [2].

*Toxoplasma* infection is usually considered self-limiting. However, it is important due to the danger of reactivation of latent infection which sometimes can be fatal in immunocompromised hosts. Furthermore, acquiring the infection during pregnancy via congenital route, particularly in the first trimester, can cause either major congenital malformations or spontaneous abortion among pregnant women who have no efficient immunity to *T. gondii* or previous exposure [3–5]. The available medications for treatment of toxoplasmosis are a combination of pyrimethamine and sulfadiazine, but the efficacy of the current therapies is limited in some cases, particularly due to significant toxicity or development of drug-resistances in parasites [6]. Additionally, the available therapies have no effect against tissue cystic stage or bradyzoites of the parasite [7, 8]. In recent years, researchers have concentrated on finding more efficacious and less toxic parasite-specific drugs against toxoplasmosis [6].

Apical end of *T. gondii* tachyzoite plays a major role during invasion and attachment process of the parasite to host cell. Moreover, some surface antigens such as perforin-like proteins assist the interaction between the tachyzoite and the host cell. This vital procedure for the parasite is accompanied by sequential secretion of three morphologically and functionally distinct organelles including micronemes, rhoptries, and dense granules. Dysfunction of any part of this essential process would be expected either to kill or inhibit the parasite invasion [9, 10].

Ketotifen (KET), a tricyclic benzocycloheptathiophene derivate, is broadly used in the control of some ailments such as allergy, asthma, and inflammatory disorders. It blocks H1 receptors, stabilizes mast cells, and inhibits eosinophil accumulation and degranulation that results in the further stabilization of the cell membrane [11, 12].

It was also shown that KET, propranolol, and cromolyn sodium act as cell membrane stabilizer drugs, and thus can inhibit penetration of *T. gondii* tachyzoite into nucleotide cells [13–15]. In continuation of our previous studies, KET was evaluated, for anti-*Toxoplasma* activity on acute phase of toxoplasmosis in a murine animal model.

## Methods

### Parasites

Tachyzoites of the highly virulent RH strain of *T. gondii* were used for experiments which routinely obtained by serial intraperitoneal (IP) passages in Balb/c female mice in Toxoplasmosis Research Center, Mazandaran University of Medical Sciences, Sari, Iran [7, 16].

### Mice

In the current study, all the experiments were conducted on female Balb/c mice weighing 18–20 g (6-week-old). All mice were housed in cages (*n* = 4) under standard laboratory conditions including an average temperature 20–25 °C, given drinking water, and regular diet [17].

### Experimental design and groups

All drugs were purchased from Sigma-Aldrich. This survey was done in pre-treatment and post-treatment groups on 48 Balb/c mice in 12 subgroups (*n* = 4) through the acute-phase of infection as previously described [7]. Briefly, in the pre-treatment study, KET both individually (2 and 3 mg/kg/day) and also in combination with PYR (50 mg/kg/day) (as the positive control) and PBS (as the negative control) was injected intraperitoneally 6, 12, and 48 h before the challenge test. In the post-treatment study, the mice were infected with 1 × 10^3^ of RH strain; then were treated 6, 12, and 48 h with the same drugs. One week after the infection, the brain biopsies of all subgroups were obtained [7]*.*

### DNA extraction and detection of *T. gondii* RE gene using Q-PCR

DNA was extracted from brain biopsies using the Viogen Tissue DNA Extraction Kit (cat. no: GG2001-50). Q-PCR was carried out using the following primers including forward primer: F 5′-AGG GAC AGA AGT CGA AGG GG-3′ and reverse primer: R 5′-GCA GCC AAG CCG GAA ACA TC-3′. Amplification of a 164 bp fragment of RE gene was performed using SYBR green master mix (Thermo Scientific cat no: K0221) (10 μl) mixed with 1.4 μl of the template DNA to reach a final volume of 20 ml containing 7 μl distilled water and 0.8 μl of each primer (1 pmol/μl). Afterward Q-PCR assays for each DNA sample was carried out in triplicate action. The number of tachyzoite in the samples was calculated as cycle threshold values (CT) based on a standard curve. The results of Q-PCR were presented as tachyzoite of *T. gondii* equivalents per mg of tissue [7]*.*

### Statistical analysis

The data were analyzed using SPSS software version 15. The comparison of data obtained with the drugs and control groups was analyzed by ANOVA and Newman-Keuls multiple comparison test. Differences were considered to be statistically significant between two groups when *P* value was less than 0.05.

## Results

In the pre-treatment group, the highest parasitic load (copy number) of *T. gondii* was observed in the negative control group (865,548 parasites/ml). In contrast, in the case group, mice brain which were treated with KET (2 mg/kg/day), showed the lowest amount of parasite load (15,698 parasites/ml) (Table 1).

**Table 1:** Anti-*Toxoplasma* activity of KET and control groups in brain tissue in the pre-treatment groups

Drug/dose, mg	CT (mean ± SD)	Parasite load	*P* value
KET			
2	28.26 ± 0.77	15,698	*P* < 0.001
3	24.53 ± 0.06	72,898	*P* < 0.001
KET and PYR			
2 + 50	23.37 ± 1.15	100,597	*P* < 0.001
3 + 50	22.42 ± 1.39	196,591	*P* < 0.001
PYR (positive control)			
50	25.28 ± 0.82	30,572	*P* < 0.001
PBS (negative control)	21.12 ± 0.34	865,548	–

In the post-treatment groups, the lowest parasitic load (13,299 parasites/ml) was observed in the combinational therapy (KET 2 mg/kg/day and PYR) (Table 2). In the pre-treatment and post-treatment groups, there was a significant difference between parasitic load in the treated and the untreated groups (negative control) (*P* < 0.001).

**Table 2:** Anti-*Toxoplasma* activity of KET and control groups in brain tissue in the post-treatment groups

Drug/dose, mg	CT (mean ± SD)	Parasite load	*P* value
KET			
2	25.40 ± 0.51	31,369	*P* < 0.001
3	25.81 ± 1.09	21,043	*P* < 0.001
KET and PYR			
2 + 50	27.26 ± 0.52	13,299	*P* < 0.001
3 + 50	26.59 ± 19.00	16,630	*P* < 0.001
PYR (positive control)			
50	25.54 ± 0.54	31,099	*P* < 0.001
PBS (negative control)	20.27 ± 0.75	1,325,000	–

**Fig. 1:**
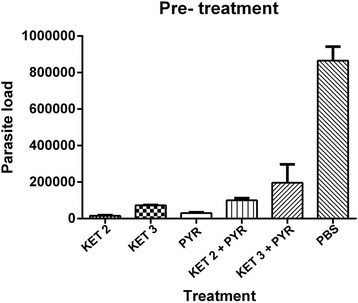
Comparison of anti-*Toxoplasma* activity of KET and the control groups in the brain tissue in the pre-treatment groups (6, 12, and 48 h before infection with 1 × 10^3^ of RH strain tachyzoites)

**Fig. 2:**
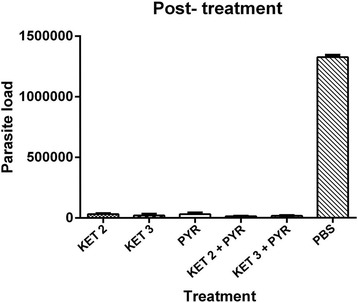
Comparison of anti-*Toxoplasma* activity of KET and the control groups in the brain tissue in the post-treatment groups (6, 12, and 48 h after infection with 1 × 10^3^ of RH strain tachyzoites)

In comparison with the negative control group most of the infected mice in other groups displayed a considerable decrease in the parasite load or circulating parasite DNA levels, in their brains (Fig. 1 and Fig. 2).

## Discussion

Standard treatment of toxoplasmosis is accompanied by significant side effects and low efficacies [6]. It was previously demonstrated that KET acts as a cell membrane stabilizer drug and thus can prevent tachyzoite of *T. gondii* from penetration into nucleated cells both in vitro and in vivo [15]. Our findings indicate that KET alone and in combination with PYR was remarkably effective against *T. gondii.*

Ryning and Remington showed that cytochalasin D inhibits the entry of *T. gondii* into peritoneal macrophages and bladder tumor cells. Microfilaments are most likely the common site of action for preventing the entrance of *T. gondii* into cells [18]. Also, Montazeri et al. reported a significant effect of propranolol against *T. gondii* infection in vivo. Propranolol as a beta-blocker, stabilizes cell membrane and inhibits parasite entry into the host cells [7, 14]. Similarly, our data demonstrated that KET individually and combined with PYR reduced the parasite load. Results indicated a remarkable effect of KET compared to a significant difference with the negative control group. However, the lowest parasitic load was observed in KET (2 mg/kg/day) in brain tissue in pre-treatment group compared to negative and positive controls. It is due to cell membrane stabilizing effect of KET.

In the post-treatment experiment, KET combined with PYR had the greatest effect in reducing the parasitic load in brain tissue. It was clearly demonstrated that KET (3 mg/kg/day) combined with PYR significantly inhibited the intracellular proliferations of the tachyzoites of highly virulent RH strain of *T. gondii.*

Martins-Duarte et al. carried out a similar study in vivo and reported that fluconazole combined with sulfadiazine and PYR was highly effective against *T. gondii* [19]. Combination therapy is known as the most effective treatment for toxoplasmic encephalitis [20]. Monotherapy administration of PYR due to the danger of reactivation and relapse of toxoplasmosis is not recommended [21].

In addition, the combined administration of KET and PYR might decrease drug dosages and consequently reduce the drug’s adverse effects. Similar effects were reported for spiramycin co-administered with metronidazole. These medications were highly efficient against the cystic form of *T. gondii* and significantly reduced the cyst load in the chronic toxoplasmosis in a mouse model [22].

Our previous studies demonstrated that the anti-*Toxoplasma* activity of propranolol combined with PYR in murine infection with the RH parasite strain in post-treatment group, significantly decreased parasitic load in brain tissue compared to the infected untreated control *(P <* 0.001*)*. Therefore, the combined administration of KET and propranolol might lead to decrease in the drug dosages and consequently drug’s side adverse effects [7, 14].

Eastman et al. indicated that KET can potently block the development of *Plasmodium falciparum* and *P. yoelii* oocysts (rodent parasite) in mosquitoes. Additionally, KET appeared to have some activities against relapse of *P. cynomolgi* infection in rhesus monkeys [23]. As there was not a significant difference in anti-*Toxoplasma* activity of drugs used in pre- and post-treatment groups, therefore, KET (2 mg/kg/day) with low dose and fewer adverse effects has treatment efficiency equal to PYR on toxoplasmosis.

## Conclusions

In conclusion, our results showed the prophylactic and therapeutic effects of KET in acute phases and that the efficiency increases in combinational therapy using both KET and PYR for the purpose of *T. gondii* inhibition in vivo. This could lead to introduction of a new drug for the treatment of toxoplasmosis, resulting in faster recovery from the disease. There is also an opportunity to decrease the dose of PYR and its related side effects using such combinations.

## Abbreviations

ARCMUMS: Animal Research Center, Mazandaran University of Medical Sciences
CT: Cycle threshold values
IP: Intraperitoneal
KET 2: Ketotifen 2 mg/kg/day
KET 3: Ketotifen 3 mg/kg/day
KET: Ketotifen
MUMSEC: Mazandaran University of Medical Sciences Ethics Committee
PBS: Phosphate-buffered saline
PYR: Pyrimethamine
Q-PCR: Quantitative-PCR
RE: Repeat element
*T. gondii*: *Toxoplasma gondii*
